# A Case Report of Sebaceous Filaments

**DOI:** 10.7759/cureus.48656

**Published:** 2023-11-11

**Authors:** Jessica P Mineroff, Jordan T Hyde, Sylvia Hsu

**Affiliations:** 1 Dermatology, State University of New York Downstate Health Sciences University, Brooklyn, USA; 2 Dermatology, Temple University Hospital, Philadelphia, USA

**Keywords:** acne vulgaris, pityriasis folliculorum, trichostasis spinulosa, trichodysplasia spinulosa, sebaceous filaments

## Abstract

This report details a case of a 16-year-old African American girl who presented with a two-year history of white spicules on her face without associated symptoms, including pruritus or pain. On physical examination, there were many 1-2 mm discreet white to yellow filamentous spicules on the mid and lower face. Histopathology scrapings showed cornified cells and calcification consistent with sebaceous filaments. Sebaceous filaments are a rare condition that presents as white-to-yellow spicules distributed in highly sebaceous areas on the face. It is caused by sebum accumulation and cornified keratinocytes surrounding hair follicles, resulting in visible excretions. Treatment of sebaceous filaments is targeted at reducing the size of sebaceous glands which subsequently decreases excretions and improves skin appearance. Despite low incidence, sebaceous filaments are likely under-reported. It is important for dermatologists to recognize and treat sebaceous filaments in patients who present with this condition to improve their appearance and quality of life. The present patient was successfully treated with topical tretinoin.

## Introduction

Sebaceous glands are acinar exocrine glands that release sebum into the hair follicle to lubricate and protect the skin. Most sebaceous glands are part of pilosebaceous units and are predominantly found on the face, scalp, chest, and upper back [1]. Sebum is made up of triglycerides, cholesterol, free fatty acids, squalene, wax esters, cholesterol esters, and diglycerides [1]. Sebum is modulated by hormones and androgens, thus sebum production peaks during puberty [2].

Sebaceous filaments are a rare condition that presents as many white-to-yellow spicules distributed in highly sebaceous areas on the face. The condition is caused by sebum accumulation, cornified keratinocytes, and dead cells that surround hair follicles and results in visible excretions [3]. The differential diagnosis for sebaceous filaments includes trichodysplasia spinulosa, trichostasis spinulosa, pityriasis folliculorum (*Demodex folliculorum*), follicular hyperkeratotic spicules (myeloma immunoglobulin or cryoglobulin), and acne vulgaris [4].

Treatment focuses on reducing the size of sebaceous glands which subsequently decreases excretions and improves skin appearance. Here, we report the case of a 16-year-old girl who presented with sebaceous filaments and was successfully treated with topical tretinoin.

## Case presentation

A 16-year-old African American girl presented with a two-year history of white papules on her face. The patient believed these papules were acne and treated her face with over-the-counter salicylic acid washes but reported little improvement. The patient denied any pruritus, pain, or other physical symptoms. However, the patient reported that the appearance of her condition was upsetting and had a negative impact on her quality of life. The patient had a history of intellectual disability. Personal and family medical history was otherwise not relevant.

**Figure 1:**
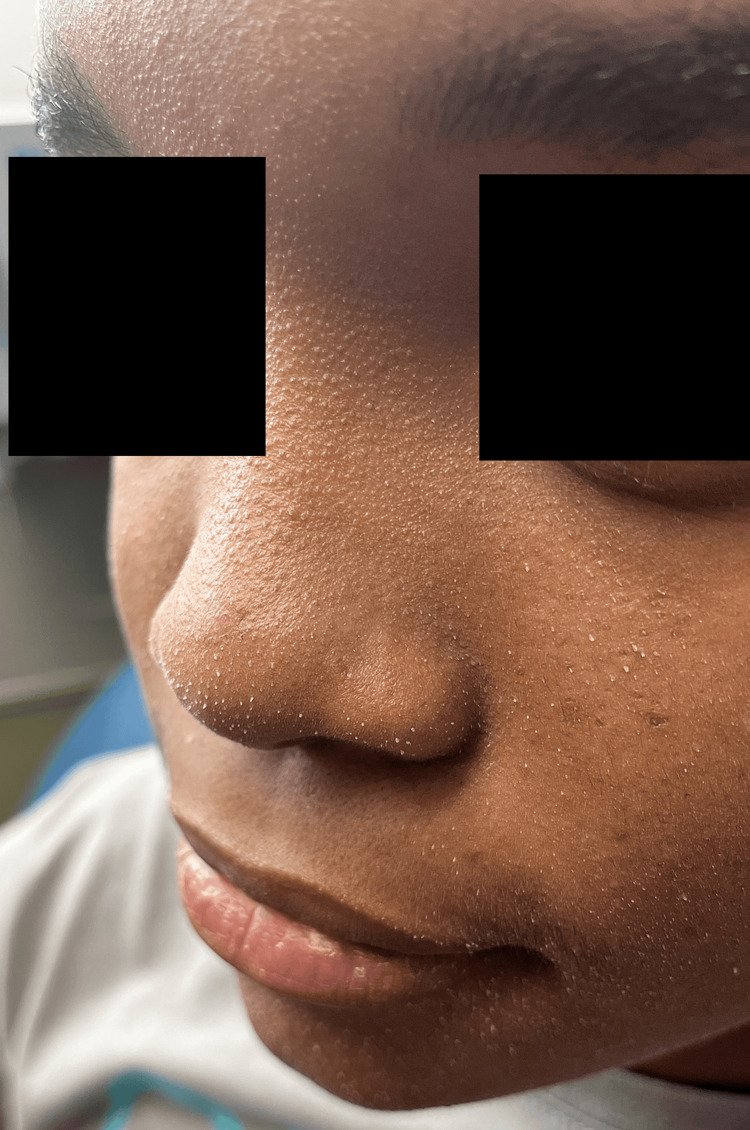
A 16-year-old girl with multiple 1-2 mm discreet white-to-yellow sebaceous filamentous spicules distributed evenly on the mid and lower face.

Physical examination revealed multiple 1-2 mm discreet white-to-yellow filamentous spicules distributed evenly on the mid and lower face (Figure 1). Some spicules were easily removed by curettage, while others required more force. The diagnosis of sebaceous filaments is made by physical examination. On histopathology, the scrapings demonstrated cornified cells and calcification consistent with sebaceous filaments. No *Demodex folliculorum* mites were seen on histopathology.

The patient was prescribed tretinoin 0.025% cream once daily. She was contacted six weeks into treatment and reported her face was clear.

## Discussion

Sebaceous filaments result from a build-up of sebum and cornified keratinocytes surrounding hair follicles [3]. Sebaceous filaments were first described in the literature in 1979 as a variant of sebaceous follicles [5]. Presentation is rare with only one other case report documenting this clinical diagnosis in a 16-year-old boy [3]. Differential diagnosis includes trichodysplasia spinulosa, trichostasis spinulosa, pityriasis folliculorum (*Demodex folliculorum*), follicular hyperkeratotic spicules (myeloma immunoglobulin or cryoglobulin), and acne vulgaris [4].

Trichodysplasia spinulosa is caused by trichodysplasia spinulosa-associated polyomavirus (TSPyV) and usually presents as erythematous papules and spicules on the face in immunosuppressed patients [6]. The prevalence of TSPyV is estimated to be as high as 65-80%; however, clinical manifestations are unique to immunocompromised patients [6]. Histology for trichodysplasia spinulosa includes keratin plugs, dilated hair follicles, and eosinophilic trichohyalin granules [6]. Diagnosis is confirmed with polymerase chain reaction, electron microscopy, or serology [6]. Treatment includes topical cidofovir and oral valganciclovir [6].

Trichostasis spinulosa is a common disorder of hair follicles that presents as darker papules on the face and extremities [7]. Diagnosis can be confirmed by dermoscopy or histology which shows hyperkeratinous sheaths surrounding vellus hairs in dilated follicles [7]. Treatments are not curative but can manage symptoms [7]. These include topical keratolytics, emollients, tretinoin, and laser treatments [7].

Pityriasis folliculorum results from infestation of the *Demodex folliculorum* mite and presents similarly to sebaceous filaments with plugged sebaceous hair follicles. The *Demodex folliculorum* mite may be in pilosebaceous units of 80-100% of patients aged 50 and over, although not all patients develop clinical symptoms [8]. In contrast to sebaceous filaments, pityriasis folliculorum can present with symptoms of burning, irritation, and pruritus [9]. Skin scraping demonstrates *Demodex folliculorum *mites [9]. Treatment includes topical metronidazole, topical ivermectin, permethrin, and benzoyl benzoate [8].

Follicular hyperkeratotic spicules are a rare disorder usually associated with underlying multiple myeloma and present as hyperkeratotic spicules on the face [10]. The diagnosis is clinical. Treatment is focused on managing the underlying multiple myeloma. Retinoids, topical keratolytics, urea, and salicylic acid can be used to manage dermatologic symptoms [10].

Sebaceous glands are involved in a range of other pathologies. Conditions arising from primary involvement include steatocystoma, sebaceous adenoma, sebaceous carcinoma, sebaceoma, sebaceous hyperplasia, and nevus sebaceous [11]. Conditions due to secondary involvement include seborrheic dermatitis and androgenic alopecia [11]. Although these conditions involve sebaceous gland pathology, their clinical presentation differs from sebaceous filaments. It is important for dermatologists to understand the structure and function of sebaceous glands and the wide range of pathologies that can result from their dysfunction to accurately diagnose and treat these conditions.

The clinical presentation of the present case favored the diagnosis of sebaceous filaments. The patient’s papules were most abundant in highly sebaceous areas, including the nose, chin, and cheeks. The patient’s adolescent age and poor hygiene due to her intellectual disability also support sebaceous filaments. The histopathology shows cornified cells and calcification further confirming sebaceous filaments. Additionally, her face cleared after topical tretinoin, which helped support the diagnosis.

Treatment for sebaceous filaments targets the sebaceous gland to reduce the size and subsequent secretions. Topical or oral retinoids, including isotretinoin, can effectively achieve this outcome and improve appearance [12]. Retinoids are vitamin A analogs that bind intranuclear retinoic acid receptors [13]. This class of medication modulates gene transcription subsequently promoting epithelial proliferation and cell turnover [13]. Retinoids inhibit the activity of sebaceous glands, decreasing sebum production [14].

## Conclusions

This case presents sebaceous filaments in a teenager who was successfully treated with topical tretinoin. The differential diagnosis for this rare condition includes trichodysplasia spinulosa, trichostasis spinulosa, pityriasis folliculorum (*Demodex folliculorum*), follicular hyperkeratotic spicules (myeloma immunoglobulin or cryoglobulin), and acne vulgaris. Despite low incidence, sebaceous filaments are likely under-reported. It is important for dermatologists to recognize and treat sebaceous filaments in patients who present with this condition to improve their appearance and quality of life.
